# Child Mortality Estimation: Estimating Sex Differences in Childhood Mortality since the 1970s

**DOI:** 10.1371/journal.pmed.1001287

**Published:** 2012-08-28

**Authors:** Cheryl Chriss Sawyer

**Affiliations:** Population Division, Department of Economic and Social Affairs, United Nations, New York, New York, United States of America; Umeå Centre for Global Health Research, Umeå University, Sweden

## Abstract

**Introduction:**

Producing estimates of infant (under age 1 y), child (age 1–4 y), and under-five (under age 5 y) mortality rates disaggregated by sex is complicated by problems with data quality and availability. Interpretation of sex differences requires nuanced analysis: girls have a biological advantage against many causes of death that may be eroded if they are disadvantaged in access to resources. Earlier studies found that girls in some regions were not experiencing the survival advantage expected at given levels of mortality. In this paper I generate new estimates of sex differences for the 1970s to the 2000s.

**Methods and Findings:**

Simple fitting methods were applied to male-to-female ratios of infant and under-five mortality rates from vital registration, surveys, and censuses. The sex ratio estimates were used to disaggregate published series of both-sexes mortality rates that were based on a larger number of sources. In many developing countries, I found that sex ratios of mortality have changed in the same direction as historically occurred in developed countries, but typically had a lower degree of female advantage for a given level of mortality. Regional average sex ratios weighted by numbers of births were found to be highly influenced by China and India, the only countries where both infant mortality and overall under-five mortality were estimated to be higher for girls than for boys in the 2000s. For the less developed regions (comprising Africa, Asia excluding Japan, Latin America/Caribbean, and Oceania excluding Australia and New Zealand), on average, boys' under-five mortality in the 2000s was about 2% higher than girls'. A number of countries were found to still experience higher mortality for girls than boys in the 1–4-y age group, with concentrations in southern Asia, northern Africa/western Asia, and western Africa. In the more developed regions (comprising Europe, northern America, Japan, Australia, and New Zealand), I found that the sex ratio of infant mortality peaked in the 1970s or 1980s and declined thereafter.

**Conclusions:**

The methods developed here pinpoint regions and countries where sex differences in mortality merit closer examination to ensure that both sexes are sharing equally in access to health resources. Further study of the distribution of causes of death in different settings will aid the interpretation of differences in survival for boys and girls.

*Please see later in the article for the Editors' Summary.*

## Abstract 2

Cheryl Sawyer uses new methods to generate estimates of sex differences in child mortality which can be used to pinpoint areas where these differences in mortality merit closer examination.

## Editors' Summary

**Background:**

In 2000, world leaders agreed to eradicate extreme poverty by 2015. To help track progress towards this global commitment, eight Millennium Development Goals (MDGs) were set. MDG 4, which aims to reduce child mortality, calls for a reduction in under-five mortality (the number of children who die before their fifth birthday) to a third of its 1990 level of 12 million by 2015. The under-five mortality rate is also denoted in the literature as U5MR and 5q0. Progress towards MDG 4 has been substantial, but with only three years left to reach it, efforts to strengthen child survival programs are intensifying. Reliable estimates of trends in childhood mortality are pivotal to these efforts. So, since 2004, the United Nations Inter-agency Group for Child Mortality Estimation (UN IGME) has used statistical regression models to produce estimates of trends in under-five mortality and infant mortality (death before age one year) from data about childbearing and child survival collected by vital registration systems (records of all births and deaths), household surveys, and censuses.

**Why Was This Study Done?:**

In addition to estimates of overall childhood mortality trends, information about sex-specific childhood mortality trends is desirable to monitor progress towards MDG 4, although the interpretation of trends in the relative mortality of girls and boys is not straightforward. Newborn girls survive better than newborn boys because they are less vulnerable to birth complications and infections and have fewer inherited abnormalities. Thus, the ratio of infant mortality among boys to infant mortality among girls is greater than one, provided both sexes have equal access to food and medical care. Beyond early infancy, girls and boys are similarly vulnerable to infections, so the sex ratio of deaths in the 1–4-year age group is generally lower than that of infant mortality. Notably, as living conditions improve in developing countries, infectious diseases become less important as causes of death. Thus, in the absence of sex-specific differences in the treatment of children, the sex ratio of childhood mortality is expected be greater than one and to increase as overall under-five mortality rates in developing countries decrease. In this study, the researcher evaluated national and regional changes in the sex ratios of childhood mortality since the 1970s to investigate whether girls and boys have equal access to medical care and other resources.

**What Did the Researcher Do and Find?:**

The researcher developed new statistical fitting methods to estimate trends in the sex ratio of mortality for infants and young children for individual countries and world regions. When considering individual countries, the researcher found that for 92 countries in less developed regions, the median sex ratio of under-five mortality increased between the 1970s and the 2000s, in line with the expected changes just described. However, the average sex ratio of under-five mortality for less developed regions, weighted according to the number of births in each country, did not increase between the 1970s and 2000s, at which time the average under-five mortality rate of boys was about 2% higher than that of girls. This discrepancy resulted from India and China—the two most populous developing countries—having sex ratios for both infant and under-five mortality that remained constant or declined over the study period and were below one in the 2000s, a result that indicates excess female mortality. In China, for example, infant mortality was found to be 12% higher for boys than for girls in the 1970s, but 24% lower for boys than for girls in the 2000s. Finally, although in the less developed regions (excluding India and China) girls went from having a slight survival disadvantage at ages 1–4 years in the 1970s, on average, to having a slight advantage in the 2000s, girls remained more likely to die than boys in this age group in several Asian and African countries.

**What Do These Findings Mean?:**

Although the quality of the available data is likely to affect the accuracy of these findings, in most developing countries the ratio of male to female under-five mortality has increased since the 1970s, in parallel with the decrease in overall childhood mortality. Notably, however, in a number of developing countries—including several each in sub-Saharan Africa, northern Africa/western Asia, and southern Asia—girls have higher mortality than boys at ages 1–4 years, and in India and China girls have higher mortality in infancy. Thus, girls are benefitting less than boys from the overall decline in childhood mortality in India, China, and some other developing countries. Further studies are needed to determine the underlying reasons for this observation. Nevertheless, the methods developed here to estimate trends in sex-specific childhood mortality pinpoint countries and regions where greater efforts should be made to ensure that both sexes have equal access to health care and other important resources during early life.

**Additional Information:**

Please access these websites via the online version of this summary at http://dx.doi.org/10.1371/journal.pmed.1001287.

- This paper is part of a collection of papers on Child Mortality Estimation Methods published in *PLOS Medicine*
- The United Nations Childrens Fund works for children's rights, survival, development, and protection around the world; it provides information on Millennium Development Goal 4, and its Childinfo website provides detailed statistics about child survival and health, including a description of the United Nations Inter-agency Group for Child Mortality Estimation; the 2011 UN IGME report Levels & Trends in Child Mortality is available
- The World Health Organization also has information about Millennium Development Goal 4 and provides estimates of child mortality rates (some information in several languages)
- Further information about the Millennium Development Goals is available
- A 2011 report by the United Nations Department of Economic and Social Affairs entitled Sex Differentials in Childhood Mortality is available

## Introduction

Sex is a key variable for disaggregation of childhood mortality rate estimates, both for monitoring and analytical purposes and as an input to other demographic estimates such as sex-specific life tables or population size and structure. However, the task of estimating trends in sex-specific child mortality, particularly for countries without reliable death registration statistics, is far from straightforward, and sex-specific data are frequently more limited or noisier than those available for both sexes combined. In this paper I outline the challenges in the estimation and interpretation of sex-specific childhood mortality rates, and develop simple methods to take advantage of available data on under-five or infant mortality by sex. The analysis updates previous United Nations work on sex differences in childhood mortality [1],[2].

### Challenges in the Estimation of Mortality Trends Disaggregated by Sex

Reliably estimating even overall trends in childhood mortality—that is, without taking into account differences by sex—is a difficult task in many developing countries. In the absence of complete and reliable vital registration systems in much of the developing world, estimation of mortality rates for children primarily relies upon data from certain questions in household sample surveys and population censuses [3]. These questions elicit information from female respondents about their childbearing history, either in detail or in summary, and the survival status of their children. Estimates based on these questions are subject to sampling errors (for surveys) and non-sampling errors (for both surveys and censuses), with the outcome that multiple inquiries may produce quite different estimates for the same time period. Since 2004, the United Nations Inter-agency Group for Child Mortality Estimation (UN IGME) has reconciled inconsistent data on overall (both-sexes) under-five mortality for each country using regression models to produce a best estimate of trends from the 1960s to the present [3]. The UN IGME also produces time series of infant mortality estimates.

One reason that the UN IGME has not taken sex-specific data into account to date is that some of the censuses and surveys on which United Nations estimates of both-sexes mortality are based did not collect the relevant data by sex. If estimates by sex were to be produced by fitting trend lines to sex-specific data, the estimates might not be consistent with estimates for both sexes combined that incorporate more data sources. A further complicating factor is that sampling error for mortality estimates from surveys—which is often large already for both sexes combined due to the relatively small number of child deaths even in a large sample—is increased when mortality estimates are disaggregated by sex or any other variable.

For countries with high-quality data from vital registration, computing mortality rates by sex from annual data is uncomplicated, but there is a further consideration pertaining to the analysis of sex differentials in mortality. In countries with low levels of mortality, ratios of male to female under-five and infant mortality can fluctuate substantially from year to year because of small numbers of deaths. For purposes of analysis and cross-national comparisons, some form of smoothing is desirable.

### Interpreting Sex Differentials in Childhood Mortality

Boys and girls have different probabilities of death due to biological factors, and these differences vary between infancy and early childhood. If sex-disaggregated estimates are to be used for monitoring or advocacy purposes, it must be clearly explained to users (1) what the expected differences are, (2) when a given difference might indicate excessive disadvantage for one sex or the other, and (3) how to understand changes.

The under-five mortality rate, also denoted in the literature as U5MR or _5_ *q*_0_, is the probability of dying between birth and exact age 5 y. The components of the under-five mortality rate examined here are the infant mortality rate (the probability of dying between birth and exact age 1 y, denoted _1_ *q*_0_) and the child mortality rate (the probability of dying between exact ages 1 and 5 y, denoted as _4_ *q*_1_). The under-five mortality rate and its components are related as follows:
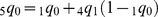
(1)The measures of sex differences employed are the ratios of male to female rates of infant, child, and under-five mortality, multiplied by 100 for ease of presentation.

**Figure 1:**
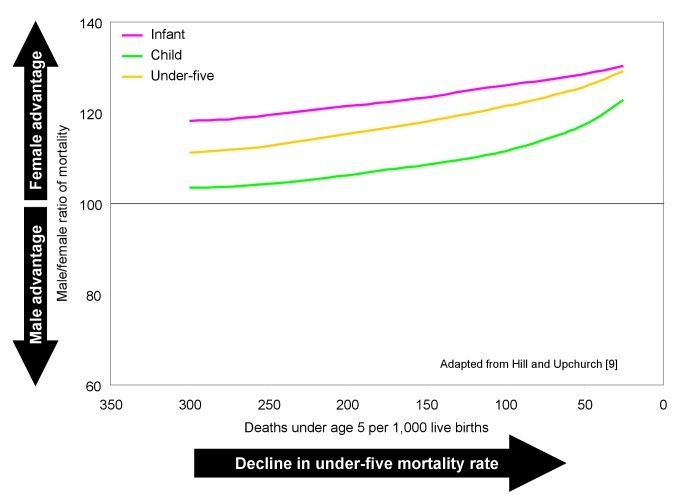
Historical change in the male-to-female ratio of mortality as under-five mortality declined in selected developed countries.

Equity in survival between females and males does not imply equal mortality rates (that is, male-to-female ratios equal to 100). Under circumstances where boys and girls have the same access to resources such as food and medical care, boys have higher mortality rates than girls during childhood, and the examined ratios would overall be expected to be greater than 100. Newborn girls have a biological advantage in survival over newborn boys, with lesser vulnerability to perinatal conditions (including birth trauma, intrauterine hypoxia and birth asphyxia, prematurity, respiratory distress syndrome, and neonatal tetanus), congenital anomalies, and such infectious diseases as intestinal infections and lower respiratory infections [4]. However, beyond early infancy, girls do not enjoy the same advantage in relation to certain infectious diseases, which are the primary causes of death in later infancy and early childhood in settings where overall mortality is high [5],[6]. Thus, the sex ratio of child mortality (that is, mortality at ages 1–4 y) is generally lower than the sex ratio of infant mortality (Figure 1). The sex ratio of under-five mortality is intermediate between the two, and will depend on the relative mortality levels of the infant and child age groups.

As living conditions improve, an “epidemiological transition” occurs during which infectious diseases recede as a cause of death [7]. As the decline in infectious disease proceeds, perinatal and congenital causes form an increasing share of total mortality among infants, while external causes, more typically affecting boys, form an increasing share of mortality for children between ages 1 and 5 y [8],[9]. Hence, as overall levels of mortality fall, female advantage in infant and child mortality would normally increase, assuming no sex-specific changes in the treatment of children. Figure 1 shows the historical change in sex ratios of infant, child, and under-five mortality for several developed countries where access of children to resources was not believed to differ greatly by sex [9]. The female advantage in survival, however, can be eroded if girls are deprived relative to boys in access to health care or to proper nutrition. If such deprivation occurs, the sex ratio of mortality might be substantially below the values shown in Figure 1 for a given level of mortality.

Because of the biologically based characteristics of differential survival by sex, it is difficult to construct a precise target of gender equity in survival in childhood. However, situations in which the survival of girls is lower than that of boys—that is, the sex ratio of mortality is less than 100—suggest that differential treatment or access to resources may be putting girls at a disadvantage. Earlier studies have found numerous countries in many regions of the world, particularly southern Asia, eastern Asia, and northern Africa/western Asia, where mortality at ages 1–4 y was higher for girls than for boys [1],[10],[11]. India and China in particular have a long-documented history of female disadvantage in mortality that is covered in an extensive literature [12]–[25].

The present study addresses the challenges outlined above in the estimation and interpretation of sex differentials. I estimate smoothed trends in the sex ratio of mortality for infants and young children to evaluate changes over time in the relative mortality of males and females for individual countries and world regions. I also examine regional differences in the relationship between the overall level of under-five mortality and sex ratios of infant and child mortality.

## Methods

### Data Sources

The three indicators of childhood mortality can be estimated directly or indirectly from demographic data sources. Direct estimates of _1_ *q*_0_, _4_ *q*_1_, and _5_ *q*_0_ are calculated from reported deaths and information on the population exposed to the risk of death, and do not require the use of models for their derivation. Direct estimates may be based either on vital events data—normally from a vital registration system or in some cases from information about household deaths collected in a census or large survey—or on survey questions posed to adult women about their childbearing histories. The childbearing histories collected in surveys comprise the date of each live birth and the age at death of children who have died before the survey date. Period-specific probabilities of dying calculated from birth histories are based on reported deaths and the numbers of children at risk of dying during a specific period, such as the 5 y preceding the survey [26].

Indirect estimates of mortality in childhood are derived from summary data on the total number of children ever born and number surviving to women of reproductive age. The method used to derive indirect estimates (also known as the Brass method) is described in detail in a United Nations manual [27]. A large number of censuses and surveys have collected the required data, although the data are not always collected or published by sex (that is, the questionnaires do not always ask about sons and daughters separately, or, if they do, the separate tabulations may not be published). The Brass method translates proportions of children surviving classified by age of mother at the time of data collection into measures of survival to various childhood ages, which in turn can be transformed into standard indicators of childhood mortality using model life tables. Under-five mortality is the preferred indicator derived via the Brass method, because _5_ *q*_0_ is more robust to the choice of model life table than _1_ *q*_0_, which can vary considerably according to the model selected. For this reason, indirect methods do not provide a satisfactory basis for estimating sex ratios of _1_ *q*_0_, since the sex ratios obtained for this indicator through indirect methods are more affected by the choice of mortality model used than are differentials in _5_ *q*_0_.

The dataset used for this study builds upon datasets [28]–[30] that were developed for a 1998 United Nations publication on sex differentials in childhood mortality [1] and expanded for a 2011 report [2] (data collection for [2] was completed in 2010; the present study incorporates additional or revised data obtained through November 2011). Microdatasets from Demographic and Health Surveys (DHS) were processed to produce a time series from each survey of direct estimates of 5-y mortality rates by sex, extending back to a period 20–24 y before each survey. In addition, tabulations of children ever born and children surviving by age of the mother were calculated by sex of the child for each DHS survey to produce indirect estimates of _5_ *q*_0_. An important new source of data since the mid-1990s is the Multiple Indicator Cluster Survey program, conducted by the United Nations Children's Fund, which has yielded additional sets of indirect estimates by sex, many for countries that had very limited data by sex from other sources. For other survey programs (including the World Fertility Survey, the Reproductive Health Survey, and the Pan Arab Project for Family Health), surveys not affiliated with the major survey programs, and censuses, the data used here are any direct or indirect estimates by sex available in published sources, or calculated from tabulations available therein. In addition, the number of data points from vital registration was greatly expanded. A large dataset of infant and under-five mortality by sex calculated from vital registration data was provided by the World Health Organization. These data were supplemented with registration data from the Human Mortality Database, the United Nations Demographic Yearbook, and other sources of life tables.

Table S1 lists the data sources considered for each country.

### Data Issues

#### Vital registration data

Data derived from the complete registration of births and deaths are the ideal basis for the estimation of mortality, since they cover the full set of events of interest and permit the estimation of trends. Unfortunately, in most developing countries the coverage and completeness of registration by vital registration systems is insufficient to produce accurate estimates of the level of childhood mortality. However, in the absence of evidence that reporting of births and deaths differs by sex of the child in a way that would affect the ratio of male to female mortality, such ratios derived from vital registration may usefully inform trends of sex differentials. The sex differentials in _1_ *q*_0_ and _5_ *q*_0_ calculated from vital registration data were used without adjustment, even when overall births and child deaths were known to be under-registered, on the assumption that under-registration in vital registration systems did not differ by sex of the child. More study is required to assess whether this assumption is valid. For most countries, however, sex differentials estimated from vital registration are consistent with those calculated from survey birth history data and often have considerably less variability. The same assumption of sex-neutral underreporting was made for data from census or survey questions on household deaths.

#### Survey data

Compared to most measures estimated by sample surveys, deaths of children are relatively rare events. The sample sizes of typical household surveys are not large enough to produce very precise estimates of childhood mortality, even for both sexes combined at the national level. In a study of 50 DHS surveys, Curtis [31] showed that relative standard error for estimated infant and under-five mortality over a 5-y period for both sexes at the national level ranged from 0.04 to 0.08, implying that the 95% confidence interval ranged from 8% to 16% on each side of the point estimate. For child mortality, relative standard errors were higher, in the range of 0.06 to 0.15, because fewer deaths occur at ages 1 to 4 y.

Such large sampling errors, which are even larger when estimates are disaggregated for a subset of the sample, complicate the assessment of trends in differential mortality by sex. For example, the male-to-female ratio of infant mortality calculated from birth histories for the Haiti 2000 DHS survey was 141 for 1991–1995 and 93 for 1996–2000, while the corresponding ratios for child mortality were 88 and 105. If taken at face value, the reported ratios would imply that the situation in Haiti changed from one in which there was excess male mortality under age 1 y and excess female mortality between ages 1 and 4 y to a reverse situation in only 5 y. The trend estimates derived in this study smooth out such fluctuations through the application of regression techniques described below.

Some important potential non-sampling biases in survey reports of childbearing histories include errors in the dating of births and deaths or omission of events from the birth history. Incorrect assignment of dates to events—for example, the heaping of date of death on 12 mo of age—can have an effect particularly on the relative levels of _1_ *q*_0_ and _4_ *q*_1_. Fortunately, for the purposes of the present study, such misdating is unlikely to occur differentially for the deaths of boys and girls, so it is unlikely to have a major impact on the sex differentials in either of these indicators. Omission of children from the birth history, on the other hand, might be more likely to differ by sex of the child. In most cases, however, there was insufficient data from alternative sources to assess whether sex-differential omission from survey birth histories was occurring. The exception was in India, where examination of sex ratios of infant mortality (SR1) from the Sample Registration System and from the National Family Health Surveys revealed systematic differences in the sex ratio of infant mortality between the two sources, with SR1 estimates from the National Family Health Surveys being consistently higher than those from the Sample Registration System. For the sex ratio of child mortality, in contrast, the two sources produced consistent estimates. The discrepancy in SR1 could be due either to defects in the sample registration system that understate male mortality, or to omission from the survey birth histories of girls who died, thus inflating survey estimates of excess male mortality. The assessment was made that the difference in SR1 was most likely due to underreporting in the National Family Health Surveys birth histories of babies who died shortly after birth, with daughters who died more likely to be omitted than sons who died [2],[32].

#### Availability of recent data

A final caveat refers to the availability of data for the 2000s. In many cases, the last available data point refers to 2005 or earlier (Table S1), and the estimates for the latter part of the decade are a projection of the earlier trend.

### Estimation Methods

The estimation of sex differentials in under-five, infant, and child mortality proceeded in three basic steps: (1) estimate trend in the sex ratio of _5_ *q*_0_ (SR5); (2) estimate, and adjust if appropriate, trend in the sex ratio of _1_ *q*_0_ (SR1); and (3) apply those trends to both-sexes estimates of _5_ *q*_0_ and _1_ *q*_0_ to derive estimate and sex ratio of _4_ *q*_1_ (SR4).

In the first step, a weighted trend line SR5*_t_* was fitted to all available SR5 estimates. The weights for data points from surveys, censuses, and vital registration were determined using a weighting scheme used in previous work by the UN IGME [33],[34]. This weighting scheme assigns progressively lower weights to direct estimates from birth histories that refer to 5-y time periods more distant from the survey date, on the assumption that recall errors may affect distant periods more strongly. For indirect data, low or zero weights are assigned to indirect estimates that are based on reports of women in the early and late childbearing years, on the assumption that these estimates may be of lower quality or subject to systematic biases.

**Figure 2:**
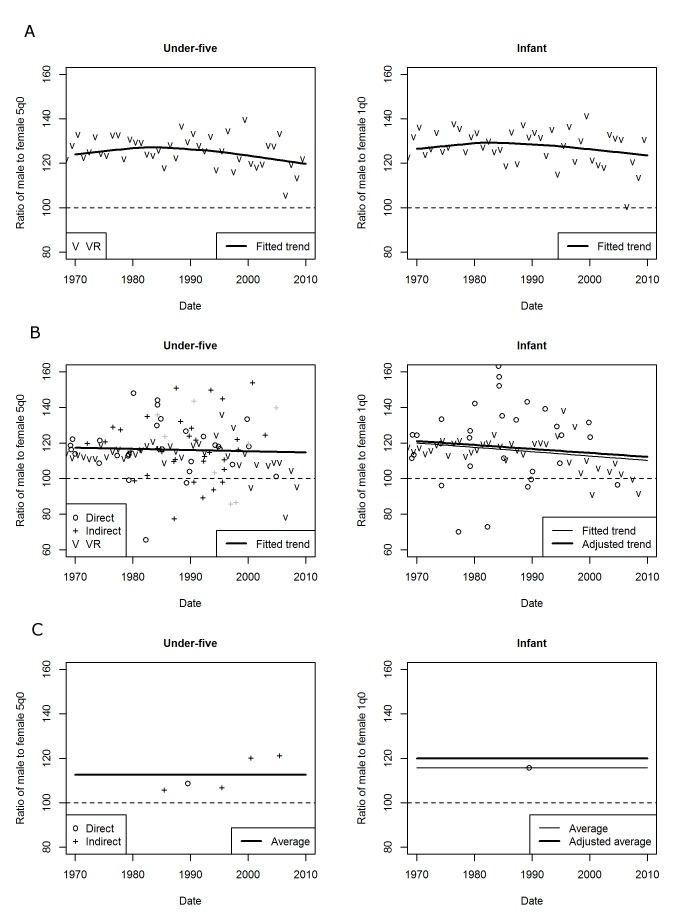
Examples of data and fits for sex ratios of under-five and infant mortality using different methods. (A) Loess method, applied to Bulgaria. (B) Linear method, applied to the Dominican Republic. (C) Average method, applied to Lao People's Democratic Republic. Points shown in gray were assigned zero weight in the weighting scheme. The fitted SR1 was adjusted in the linear and average methods to account for the exclusion of indirect data. VR, vital registration.

Because of variations between countries in the amount and consistency of data available, three different methods were ultimately employed to estimate sex ratios of mortality. Initially, loess regression was tested for all countries. The loess method fits a series of polynomials to localized subsets of the data centered on each point of the dataset. The weight of each data point in the localized regression is determined by its distance from the center. A bandwidth, denoted alpha, selected by the user, determines the proportion of the dataset used to fit each local regression. A number of different alpha values were tested, to impose varying degrees of smoothing. For countries where estimates were based on a time series of vital registration data, it was found that the loess with an alpha of 0.75 captured changes in trend without being overly sensitive to short-term variation. In addition, a re-descending M estimator with Tukey's biweight function was applied in the loess procedure in R (family = “symmetric”) to reduce the influence of more extreme data points. The case of Bulgaria is shown in Figure 2A to illustrate the loess fitting method.

For countries where the primary sources of data were sample surveys, however, the degree of smoothing required to achieve plausible results with the loess often was so strong that the results differed little from a simpler linear regression. The linear regression line had the additional benefit of being more straightforward to adjust for SR1, as described below. Therefore, for many developing countries the results are based on robust linear regression (implemented with the rlm function in the R MASS package and hereafter referred to as the “linear method”), shown for the Dominican Republic in Figure 2B.

There were a number of countries where neither loess nor robust linear regression fitted to all data points was able to produce a result that was satisfactory for purposes of analysis or disaggregation. In a few of these countries, where time series of vital registration data were available to fit a stable trend and survey data had high sampling error, the decision was made to exclude the survey data and fit the loess or robust linear regression to the vital registration data only. In countries where such a stable time series was not available, a simple weighted average of all available SR5 data was computed (as in Figure 2C for Lao People's Democratic Republic). Such an average may be useful for disaggregating both-sexes estimates if no other method is available, but it does not give any information on time trends in SR5. For this reason, results from the average method are not analyzed at length, and countries where it was used are excluded from the time trend analysis for regional aggregations presented below. Table S2 indicates which of the methods—loess, linear, or average—was used to produce estimates of sex differentials for each country.

The second step of the estimation process was to fit a trend line SR1*_t_* to available data on SR1. As was noted in the previous section on data sources, indirect data on SR1 were not included in the analysis because SR1 is less robust than SR5 to the choice of model life table. Yet, using only direct data for SR1 while using both direct and indirect data for SR5 could cause inconsistency between time series fitted for SR1 and SR5. This was particularly the case in countries where a number of surveys had collected indirect data only. Therefore, in order to best exploit the available information, direct and indirect estimates of SR5 were used to adjust direct estimates of SR1 in the linear and average methods. A preliminary trend line, SR1_t_*, was fitted to SR1 from direct data using the same fitting method that had been selected for SR5*_t_*. If no indirect data had been used to fit SR5*_t_*, then SR1_t_* was adopted as the final estimate SR1*_t_*. If both direct and indirect data had been used to fit SR5*_t_*, an additional trend line, SR5_t_*, was fitted to SR5 points coming from direct data only. The ratio of SR5*_t_*/SR5_t_* was used to adjust SR1_t_*, producing the final estimate SR1*_t_*. Figure 2B and 2C show the results of this adjustment for the Dominican Republic and Lao People's Democratic Republic, respectively.

Predicted SR5*_t_* and SR1*_t_* were applied to estimates of _5_ *q*_0_ and _1_ *q*_0_ for both sexes to produce time series of infant and under-five mortality levels by sex. Levels of _5_ *q*_0_ by sex for time period *t* were derived from the both-sexes estimates using the formulas
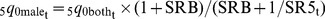
(2)and
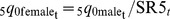
(3)where SRB is the sex ratio at birth as estimated for each country for the period 2000–2005 in *World Population Prospects: The 2010 Revision* [35]. Corresponding formulas were applied for infant mortality. Then, _4_ *q*_1male_ and _4_ *q*_1female_ were derived via the relationship in Equation 1. The resulting _4_ *q*_1male_ and _4_ *q*_1female_ were used to compute estimates of SR4*_t_*. These derived estimates of SR4*_t_* were compared to direct data on SR4 from surveys or vital registration and generally found to be consistent.

Estimates of under-five and infant mortality rates for both sexes combined were taken from two United Nations sources, *World Population Prospects: The 2010 Revision*, produced by the Population Division [35], and *Levels & Trends in Child Mortality: Report 2011*, produced by the UN IGME [36],[37]. The estimates from these two sources are generally quite similar for _5_ *q*_0_—the indicator coordinated by the UN IGME—but can differ somewhat more for _1_ *q*_0_, usually because of the use of different model life tables. The both-sexes estimates for _5_ *q*_0_ and _1_ *q*_0_ from *World Population Prospects* [35] were used in this report for most developing countries (noting that for _5_ *q*_0_ the estimates referring to periods prior to 1980 are unpublished). The choice of which series of both-sexes estimates to use does not affect the estimated trends SR5*_t_* or SR1*_t_*, as those come from the data, but it does affect estimated trends in SR4*_t_* because the trend in the sex ratio of _4_ *q*_1_ derived from estimated _5_ *q*_0_ and _1_ *q*_0_ is dependent on the relative levels of _5_ *q*_0_ and _1_ *q*_0_ as well as the sex differentials in each. There were only a few cases where the choice of both-sexes estimate made an appreciable difference in SR4*_t_*. For countries of the more developed regions, estimates from *Levels & Trends in Child Mortality* [36] were used because levels of _5_ *q*_0_ and _1_ *q*_0_ from this set of estimates are taken directly from annual vital registration.

For countries where averages of SR5 and SR1 were employed, these average ratios were applied to the whole series of both-sexes estimates. It should be noted that applying constant SR5*_t_* and SR1*_t_* to changing both-sexes estimates results in SR4*_t_* values that change over time. However, these changes in SR4*_t_* should not be interpreted as trends and will not be presented as such. As noted above, estimates for countries where the average method was used are not included in the aggregated trends for regions and development groups presented below.

Estimates were attempted for all countries or areas (hereafter referred to as countries) that had a population of 1 million or more in 2010. Out of the 156 countries with such a population, estimates were generated for 153 countries (Table 1). Of these, 113 were in less developed regions, comprising Africa, Asia excluding Japan, Latin America/Caribbean, and Oceania excluding Australia and New Zealand (the lists of countries located in the less developed regions and more developed regions as well as the other geographical groupings used for this study—sub-Saharan Africa, northern Africa/western Asia, eastern/southeastern Asia, Commonwealth of Independent States [CIS] Asia, Latin America/Caribbean, and developing Oceania—are shown in Table S3). Ninety-two countries in the less developed regions, containing 92% of the population of those regions, had sufficient data to apply the methods developed for trend analysis. For an additional 21 countries, holding 6% of the population of the less developed regions, enough data were available to estimate average sex differentials in under-five or infant mortality, which were assumed to apply to the entire time span under consideration.

**Table 1:** Number of countries or areas and percentage of population covered in the study.

Measure	Region or Development Group	Grand Total	Included			Not Included	
			Using Trend Estimate Method	Using Average Method	Total	Insufficient Data	Less than 1 Million Population in 2010
**Number of countries or areas**	**World**	229	131	22	153	3	73
	**Less developed regions**	173	92	21	113	3	57
	Sub-Saharan Africa	49	32	9	41	0	8
	Northern Africa/western Asia	23	13	5	18	3	2
	Eastern/southeastern Asia (excluding Japan)	17	11	4	15	0	2
	Southern Asia	9	5	2	7	0	2
	CIS Asia	8	8	0	8	0	0
	Latin America/Caribbean	46	23	0	23	0	23
	Developing Oceania (excluding Australia and New Zealand)	21	0	1	1	0	20
	**More developed regions**	56	39	1	40	0	16
**Population in 2010 (thousands)**	**World**	6,895,889	6,452,879	368,629	6,821,508	57,674	16,706
	**Less developed regions**	5,659,989	5,223,246	364,869	5,588,115	57,674	14,200
	Sub-Saharan Africa	811,887	761,961	46,688	808,649	0	3,237
	Northern Africa/western Asia	425,248	350,255	39,578	389,833	34,458	956
	Eastern/southeastern Asia (excluding Japan)	2,040,849	1,850,332	166,359	2,016,691	23,216	943
	Southern Asia	1,704,146	1,597,719	105,385	1,703,105	0	1,042
	CIS Asia	77,358	77,358	0	77,358	0	0
	Latin America/Caribbean	590,024	585,621	0	585,621	0	4,403
	Developing Oceania (excluding Australia and New Zealand)	10,477	0	6,858	6,858	0	3,619
	**More developed regions**	1,235,900	1,229,633	3,760	1,233,394	0	2,506
**Percentage of population**	**World**		93.6	5.3	98.9	0.8	0.2
	**Less developed regions**		92.3	6.4	98.7	1.0	0.3
	Sub-Saharan Africa		93.9	5.8	99.6	0.0	0.4
	Northern Africa/western Asia		82.4	9.3	91.7	8.1	0.2
	Eastern/southeastern Asia (excluding Japan)		90.7	8.2	98.8	1.1	0.0
	Southern Asia		93.8	6.2	99.9	0.0	0.1
	CIS Asia		100.0	0.0	100.0	0.0	0.0
	Latin America/Caribbean		99.3	0.0	99.3	0.0	0.7
	Developing Oceania (excluding Australia and New Zealand)		0.0	65.5	65.5	0.0	34.5
	**More developed regions**		99.5	0.3	99.8	0.0	0.2

Trends were estimated for 39 countries in the more developed regions (comprising Europe, northern America, Japan, Australia, and New Zealand), while for one developed country (Bosnia and Herzegovina), only average sex differentials could be estimated.

The methods and results presented in this article were developed as an analytical study separate from the production of United Nations mortality estimates published in *Levels & Trends in Child Mortality* [36] or *World Population Prospects* [35]. The mortality estimates by sex presented here may differ from estimates in forthcoming editions of those publications due to differences in data availability or other methodological considerations.

## Results

Trends in the sex ratios of under-five, infant, and child mortality are summarized as decade averages for the 1970s, 1980s, 1990s, and 2000s. Two different approaches to examining global and regional trends are examined: first, considering each country as a unit of analysis for computing median country-specific ratios (Table 2) and, second, weighting country mortality rates by number of births to produce weighted regional averages (Table 3). The country results on which the regional analyses are based may be found in Table S2. Only the 131 countries for which trend estimates were produced were included in the computation of regional medians and averages.

**Table 2:** Median sex ratios of infant, child, and under-five mortality by region, 1970s–2000s.

Region or Development Group	Number of Countries with Trend Estimates	Median Ratio of Male to Female Mortality (per 100)														
		Infant Mortality					Child Mortality					Under-Five Mortality				
		1970s^a^	1980s	1990s	2000s	Change from 1970s to 2000s	1970s^a^	1980s	1990s	2000s	Change from 1970s to 2000s	1970s^a^	1980s	1990s	2000s	Change from 1970s to 2000s
**World**	131	121	122	122	121	0	106	109	111	116	9	115	117	119	120	5
**Less developed regions**	92	118	118	119	120	2	100	103	107	112	12	111	113	114	117	6
Sub-Saharan Africa	32	115	116	117	117	2	102	103	105	107	5	109	111	111	113	4
Northern Africa/western Asia	13	112	115	117	116	5	94	99	105	122	28	107	109	113	116	10
Eastern/southeastern Asia	11	124	124	123	121	−3	100	106	110	118	18	116	117	117	117	1
Southern Asia	5	111	112	113	114	3	84	83	82	93	9	101	101	102	106	4
CIS Asia	8	—	124	127	131	3	—	103	107	109	2	—	118	123	126	3
Latin America/Caribbean	23	122	122	123	122	1	106	111	114	117	11	117	120	121	122	5
**More developed regions**	39	129	129	126	123	−6	125	126	124	124	−1	128	128	125	123	−6

a Estimates for the 1970s exclude the following countries that are included for subsequent decades: Albania, Armenia, Azerbaijan, Belarus, Croatia, Czech Republic, Estonia, Georgia, Kazakhstan, Kyrgyzstan, Latvia, Lithuania, Mongolia, Qatar, Republic of Moldova, Serbia, Sierra Leone, Slovakia, Slovenia, Somalia, Tajikistan, the former Yugoslav Republic of Macedonia, Timor-Leste, and Turkmenistan.

**Table 3:** Regional average estimates of male, female, and both-sexes infant, child, and under-five mortality, and sex ratios of infant, child, and under-five mortality, 1970s–2000s.

Region or Development Group	Decade^a^	Infant Mortality Rate (Deaths under Age 1 y per 1,000 Live Births)			Child Mortality Rate (Deaths at Ages 1–4 y, per 1,000)			Under-Five Mortality Rate (Deaths under Age 5 y, per 1,000 Live Births)			Ratio of Male to Female Mortality (per 100)		
		Male	Female	Both Sexes	Male	Female	Both Sexes	Male	Female	Both Sexes	Infant Mortality	Child Mortality	Under-Five Mortality
**World**	1970s	85	77	81	45	50	47	125	122	123	111	90	103
	1980s	70	64	67	32	37	34	99	97	98	110	89	102
	1990s	60	56	58	28	30	29	85	84	84	107	91	101
	2000s	49	46	47	22	24	23	69	68	69	107	94	103
**World excluding China and India**	1970s	91	77	84	47	49	48	132	120	126	118	98	110
	1980s	74	63	69	37	38	38	107	98	103	117	98	110
	1990s	65	55	60	34	34	34	95	86	91	117	101	111
	2000s	53	46	49	27	26	27	78	70	74	117	103	111
**Development group**													
Less developed regions	1970s	95	86	91	51	57	54	141	138	139	110	90	102
	1980s	78	71	75	37	42	39	111	109	110	109	88	102
	1990s	66	62	64	31	34	32	94	93	93	107	90	101
	2000s	54	51	53	25	26	26	77	75	76	107	94	102
Less developed regions excluding China and India	1970s	112	95	104	61	63	62	165	151	158	118	97	109
	1980s	91	77	84	47	48	47	132	120	126	117	98	110
	1990s	77	66	72	41	41	41	114	103	108	117	101	110
	2000s	63	54	58	33	32	32	92	83	88	117	103	111
More developed regions	1970s	22	17	19	4.2	3.5	3.8	26	20	23	129	122	127
	1980s	16	12	14	3.4	2.7	3.1	19	15	17	129	124	128
	1990s	11	8.4	9.6	2.3	1.8	2.1	13	10	12	127	125	127
	2000s	7.5	6.0	6.7	1.6	1.3	1.4	9.0	7.2	8.1	125	124	124
**Region**													
Sub-Saharan Africa	1970s	134	115	125	100	98	99	219	201	210	116	102	109
	1980s	122	105	114	87	85	86	198	180	189	116	103	110
	1990s	115	98	107	81	78	79	185	169	177	116	103	110
	2000s	97	83	90	63	61	62	153	139	146	116	103	110
Northern Africa/western Asia	1970s	125	113	119	51	56	54	169	162	166	111	91	105
	1980s	87	78	82	33	35	34	117	110	114	112	94	107
	1990s	62	54	58	22	22	22	82	74	78	114	99	110
	2000s	42	36	39	13	13	13	54	48	51	117	104	113
Eastern/southeastern Asia	1970s	57	49	53	33	34	34	89	81	85	118	97	109
	1980s	42	39	41	18	19	18	59	57	58	108	93	103
	1990s	30	33	31	9.8	10	9.9	40	42	41	92	98	94
	2000s	23	26	24	6.1	5.7	5.9	29	31	30	91	107	94
Eastern/southeastern Asia excluding China	1970s	84	66	75	36	36	36	116	99	108	126	99	117
	1980s	59	47	53	22	22	22	79	68	74	124	102	117
	1990s	41	33	37	13	12	13	53	45	49	123	107	119
	2000s	29	24	27	8.5	7.4	8.0	37	31	34	122	115	120
Southern Asia	1970s	117	116	117	55	74	64	166	181	173	101	75	92
	1980s	95	92	93	37	52	44	129	138	133	103	73	93
	1990s	76	74	75	28	38	33	102	109	105	103	75	94
	2000s	58	58	58	20	25	22	77	81	79	101	79	95
Southern Asia excluding India	1970s	140	122	131	70	85	78	200	196	198	115	82	102
	1980s	110	96	103	48	58	53	153	148	151	114	83	103
	1990s	87	77	82	33	37	35	117	111	114	113	89	105
	2000s	65	58	62	21	21	21	85	78	81	113	100	109
CIS Asia	1970s	—	—	—	—	—	—	—	—	—	—	—	—
	1980s	79	62	71	18	17	18	95	78	87	128	102	122
	1990s	67	51	59	15	14	15	81	65	73	130	106	125
	2000s	50	38	44	11	10	10	60	47	54	131	110	127
Latin America/Caribbean	1970s	83	68	76	34	33	33	114	98	106	122	105	116
	1980s	58	47	53	17	16	17	74	63	68	123	105	118
	1990s	39	32	35	11	10	11	50	42	46	123	109	120
	2000s	26	21	24	6.9	6.1	6.5	33	27	30	123	113	121
**Selected countries**													
China	1970s	47	42	44	32	34	33	78	74	76	112	96	105
	1980s	36	36	36	16	18	17	51	53	52	99	90	96
	1990s	26	33	29	8.3	8.8	8.5	34	41	37	79	94	82
	2000s	20	27	23	4.8	4.7	4.8	25	31	28	76	102	80
India	1970s	110	114	112	50	70	60	155	176	165	96	72	88
	1980s	90	90	90	34	49	41	121	135	128	100	69	89
	1990s	73	73	73	26	38	32	97	108	102	100	70	90
	2000s	56	58	57	19	26	23	74	82	78	97	74	90

Countries weighted by number of births.

a Estimates for the 1970s exclude the following countries that are included for subsequent decades: Albania, Armenia, Azerbaijan, Belarus, Croatia, Czech Republic, Estonia, Georgia, Kazakhstan, Kyrgyzstan, Latvia, Lithuania, Mongolia, Qatar, Republic of Moldova, Serbia, Sierra Leone, Slovakia, Slovenia, Somalia, Tajikistan, the former Yugoslav Republic of Macedonia, Timor-Leste, and Turkmenistan.

Table 2 shows that in the less developed regions the median sex ratio of under-five mortality increased from the 1970s to the 2000s. For the 92 countries in the less developed regions for which trends were estimated in this study, the median sex ratio of under-five mortality increased from 111 in the 1970s to 117 in the first decade of the 2000s. Thus, in the majority of developing countries, females have an advantage in survival to age 5 y, and this advantage has increased, as expected from the historical experience of developed countries as described above, as mortality has declined. This increase is due primarily to increases in the sex ratio of mortality at ages 1–4 y in many countries, while increases in the sex ratio of the infant component of under-five mortality have been smaller.

**Figure 3:**
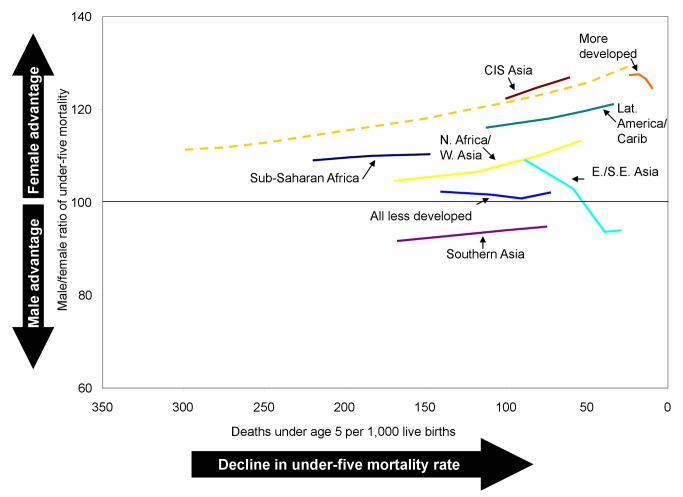
Trends in the male-to-female ratio of under-five mortality by level of under-five mortality. Dashed line is the historical sex ratio of under-five mortality for selected developed countries from Hill and Upchurch [9].

However, when countries are weighted according to the number of births, no such rise in the sex ratio of under-five mortality is seen. On average, the sex ratio of under-five mortality in the less developed regions remained nearly constant, around 101 to 102, from the 1970s to the 2000s (Table 3; Figure 3). This difference between the median trend and the birth-weighted trend occurs because the sex ratios of under-five mortality estimated for the two most populous countries, China and India, constitute important exceptions to the rising trend. Estimated sex ratios of under-five mortality for the first decade of the 2000s were below 100 in both countries (Table 3), indicating substantial excess female mortality. In China, the sex ratio of under-five mortality declined between the 1970s and the 2000s, while in India it remained roughly constant, suggesting that even though mortality rates were falling in both countries, girls did not share in survival improvements to the expected extent.

Moreover, China and India were the only two countries in the world where female infant mortality was higher than male infant mortality in the 2000s. In China, the sex ratio of infant mortality fell from 112 in the 1970s to 76 in the 2000s (Table 3), that is, from a situation where infant mortality was 12% higher for boys than for girls to one where infant mortality was 24% lower for boys.

In India, female infant mortality was roughly equal to or slightly higher than male infant mortality throughout the decades examined, but girls' survival disadvantage was particularly acute in the 1–4-y age group. In the 2000s, the ratio of male to female child mortality was estimated at 74 (Table 3), meaning that girls' mortality between ages 1 and 5 y was more than 30% higher than boys'. While the estimates suggest that the sex ratio of child mortality may have increased somewhat in India since the 1980s, girls remain disadvantaged in mortality, compared both to the sex differences found in other parts of the developing world and to the historical experience of developed countries at the same level of mortality.

The lower relative survival of girls to age 5 y in China and India has a large impact on estimates of average sex differentials for their respective regions of Asia, as well as on the average for the less developed regions. The average sex ratio of under-five mortality for eastern and southeastern Asia declined from 109 in the 1970s to 94 in the 2000s (Table 3). However, the average for the countries of the region apart from China rose from 117 to 120. The sex ratio of under-five mortality in southern Asia rose slightly from 92 to 95, but increased more steeply, from 102 to 109, in the countries of the region other than India. The estimates in this study suggest that the survival disadvantage of girls has lessened more in other countries of southern Asia than in India, with the exception of Nepal.

**Figure 4:**
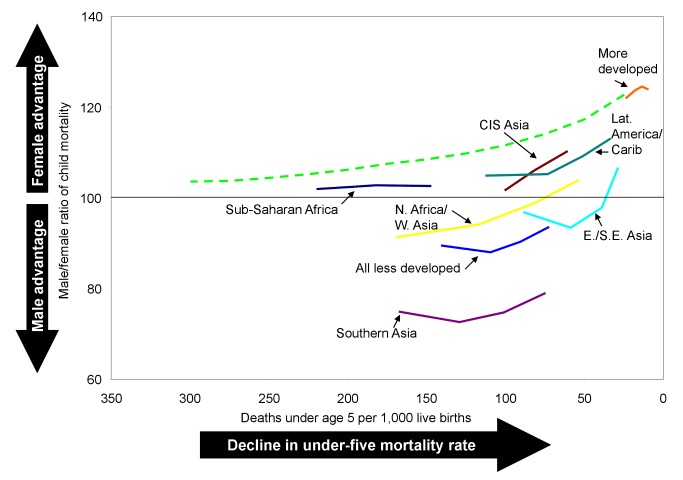
Trends in the male-to-female ratio of child mortality (ages 1–4 y) by level of under-five mortality. Dashed line is the historical sex ratio of child mortality for selected developed countries from Hill and Upchurch [9].

In many of the less developed regions, girls' past disadvantage in mortality at ages 1–4 y appears to be easing. The regions of northern Africa/western Asia, eastern/southeastern Asia, southern Asia, CIS Asia, and Latin America/Caribbean all experienced increases in the average sex ratio of child mortality of 6 or more percentage points (Table 3). In sub-Saharan Africa, however, there was essentially no change in the average sex differential of child mortality, with increasing ratios in many countries offset by decreasing ratios in others. For the less developed regions on average (excluding China and India), girls went from a situation of slight disadvantage in mortality at ages 1–4 y in the 1970s to a slight advantage in the 2000s. However, the average ratios of child mortality in all regions of the developing world remain below those expected based on the historical experience of some developed countries at similar levels of mortality (Figure 4).

**Figure 5:**
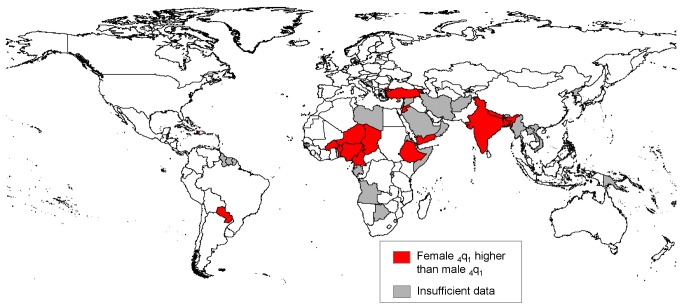
Countries where excess female child mortality (ages 1–4 y) was found in the 2000s.

The rising regional average sex ratios of child mortality mask a number of cases where the estimates suggest continued or worsening female disadvantage in mortality at ages 1–4 y. While the case of India was highlighted above because of its weight in regional and world averages, there are many other countries where mortality in this age group was higher for girls than for boys in the 2000s. The countries where excess female child mortality was apparent in the 2000s are indicated in Figure 5. While countries with excess female mortality can be found in most regions of the developing world, there are notable concentrations in southern Asia and in the western and middle regions of sub-Saharan Africa, as well as several countries in northern Africa/western Asia. While data quality issues may affect the reliability of these estimates, countries with apparent female disadvantage merit further study to see if differential treatment is an issue.

**Figure 6:**
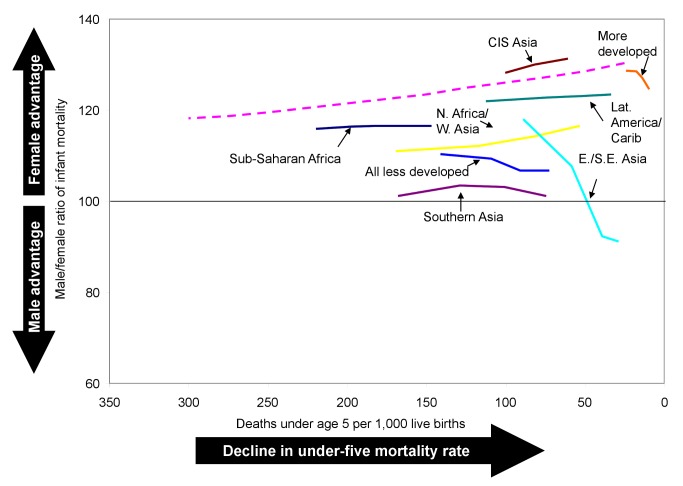
Trends in the male-to-female ratio of infant mortality by level of under-five mortality. Dashed line is the historical sex ratio of infant mortality for selected developed countries from Hill and Upchurch [9].

Among infants under age 1 y, girls continue to have the advantage in survival in all countries apart from China and India. However, the female survival advantage in infancy in most of the developing world is not as great as would be expected based on the historical experience of some developed countries at similar levels of mortality (Figure 6). It cannot be stated with certainty whether this finding is due to differences in the treatment of girls and boys, to factors such as differences in cause-of-death patterns or the rollout of medical interventions in different locations at a given level of mortality, or to issues with the quality of the data for some countries that affect the estimates in a systematic way.

In the more developed regions, ratios of male to female infant mortality have been declining in recent decades (Tables 2 and 3; Figure 6), meaning that the male disadvantage in infant mortality is becoming smaller. This trend started in certain countries in the 1970s and has since spread to almost all of the developed countries and to a number of countries in the less developed regions that have relatively low levels of mortality. The change in trend may be attributable to improvements in neonatal care that have decreased deaths from prematurity and respiratory distress, causes that have a greater impact on male infants [38]. However, further study is required to confirm the causes of this trend.

**Figure 7:**
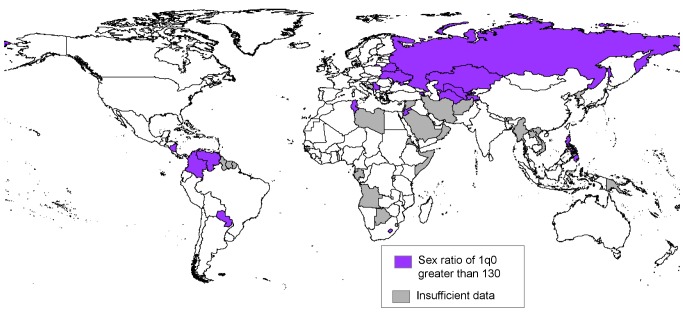
Countries where excess male infant mortality was found in the 2000s.

Several countries had findings of unusually high sex ratios of infant mortality (greater than 130), suggesting a greater than expected degree of male disadvantage in survival. These countries, found in both more developed and less developed regions, include many of the European and Asian countries of the former Union of Soviet Socialist Republics (Figure 7). The high ratios could be due to lack of access to the advances in medical care that have led to declining sex ratios of infant mortality in most of the more developed regions, but again, more detailed examination of causes of infant deaths by age and sex is required.

## Discussion

Our estimates of long-term trends in the sex ratios of infant, child, and under-five mortality show that in the majority of countries in the less developed regions, the ratio of male to female under-five mortality has increased since the 1970s. This is due primarily to increases in the sex ratio of mortality at ages 1–4 y, while changes in the sex ratio of infant mortality have been smaller. There remain, however, a number of developing countries where girls have higher mortality than boys at ages 1–4 y, with concentrations in middle and western sub-Saharan Africa, northern Africa/western Asia, and southern Asia. Estimated infant mortality was higher for girls than for boys in only two countries, India and China. Meanwhile, in the more developed regions, a reversal of the historically rising trend in sex ratios of infant mortality has been observed as countries approach very low levels of mortality.

Estimates of under-five mortality levels are receiving intense focus as the world nears the 2015 target date for the Millennium Development Goals. The target for Millennium Development Goal 4 calls for reducing under-five mortality by two-thirds from its 1990 level, and efforts to strengthen child survival programs are intensifying. In many areas of the world, advances in survival appear to be accruing relatively equitably to girls and boys, in line with the changes in sex differentials expected given the changing cause-of-death patterns that accompany mortality decline. However, this is not universally the case. Findings of low or declining sex ratios of infant or child mortality in a number of countries that still have relatively high mortality may merit concern, as they suggest that girls in these countries may not be sharing fully in the recent improvements in survival. Further study is needed to confirm these findings, to identify why girls' relative survival may not be keeping pace in some countries, and to assess interactions with other barriers to care such as poverty or marginalization.

### Studying Regions with Excess Female Mortality

Regions where concentrations of excess female child mortality were found would benefit from in-depth cross-national or sub-national studies of cause-specific mortality and mortality determinants by sex. Regions such as middle and western sub-Saharan Africa, northern Africa/western Asia, and southern Asia each have a number of countries with excess female child mortality, but also have countries where mortality is (or has become) higher for boys. Case studies from countries that have been successful in reducing inequalities in the survival of girls and boys—whether this was a conscious policy choice or an indirect outcome of generally expanded access to interventions—could provide useful insights and guidance for the planning of child health interventions and health system improvements.

The situation of girls in China and India, already well documented in the literature, merits continued study, as there is evidence that girls are not benefiting as much as boys from the mortality declines in these countries. The interaction of strong son preference and declining fertility has continued implications for the health and survival of girls in these countries. Both countries have implemented policies and programs intended to improve the status of girls and women as well as directly influence families' treatment of girls [2], but the available data, which refer most recently to 2005 for China and 2009 for India (Table S1), do not indicate significant change as yet in girls' relative survival to age 5 y, and trends in these countries should be reassessed as new data become available. In both countries, media and policy attention in recent years have concentrated largely on sex-selective abortion—that is, prenatal discrimination—but differences in postnatal treatment still have mortality consequences for large numbers of girls, particularly in India, where relatively high infant and child mortality rates mean that a significant number of excess deaths still occur.

The methods presented here have the benefit of producing comparable results for countries in a wide variety of data situations. They are valuable for making sense of noisy data from the often patchy collection of survey, census, and vital registration sources for many developing countries. Yet, they are also useful for analyzing sex differentials even in situations of good data quality and when mortality is low, because year-to-year fluctuations in the sex ratio of mortality can be substantial when numbers of infant and child deaths are low, as is the case even in very large countries with low mortality levels.

### Limitations

The methods are subject to a number of limitations. Despite the use of robust regression methods to limit the influence of extreme data points that are due to sampling error, the nature of the data—which for many countries can be variable, sparse, or from sources for which data collection quality cannot be adequately assessed—does not permit strong conclusions. Particularly in the case of countries with only a few sources of data, addition of a new source may change the results substantially. Future work should aim to quantify the uncertainty of the estimates.

Another limitation, from an analytical standpoint, is that sex differentials in overall under-five mortality cannot be explained well without a nuanced understanding of mortality in the component age groups. The reliance on the sex ratio of _4_ *q*_1_ as an important indicator of sex differentials in this study is somewhat problematic. This ratio is calculated from sex-specific estimates of _4_ *q*_1_ that are derived after fitted trends in the sex ratios of infant and under-five mortality are used to disaggregate both-sexes estimates of infant and under-five mortality. Thus, the sex ratio of _4_ *q*_1_ is sensitive both to the fitted sex ratios of infant and under-five mortality, and to the relative levels of both-sexes infant and under-five mortality used as inputs. However, in cases where direct data on _4_ *q*_1_ by sex were available for comparison, the derived trends in the sex ratio of _4_ *q*_1_ were generally consistent with the empirical trends.

The estimates derived here will be useful for incorporation into life tables for the estimation of mortality and population change from the 1970s until today. In this way, they represent an advance over earlier studies of sex differentials in child mortality. However, the usefulness of the fitted trends for the projection of sex differentials in individual countries may be limited, particularly in cases where the estimated increases or decreases are steep and such rapid change cannot sensibly be projected into the future.

### Conclusions

The results obtained here could be a first step in developing a model based on the experience of countries with low mortality, or on regional trends, to blend with the estimates, for purposes of projection. Such a model will also be useful for countries with little or no information on which to base estimates of sex differentials in mortality. However, I believe there is merit in using country-specific data to the extent possible, especially if the intent is to have policy-relevant monitoring of sex differences in mortality. Models based strictly on levels of mortality or regional dummies could mask findings that should prompt further investigation, such as the different trends in sex differentials among countries within regions such as northern Africa/western Asia and western Africa.

Despite the limitations detailed above, the methods developed here may be useful for the international community. The methods can be easily implemented by researchers or national authorities working with standard registration or survey data in their own countries. Applied cross-nationally, the methods can advance understanding of the dynamics of childhood mortality by sex around the world.

## Supporting Information

Table S1 **Data sources.**
(XLS)

Table S2 **Sex ratios and levels of infant, child, and under-five mortality for countries, 1970s–2000s.**
(XLS)

Table S3 **Regional groupings used in the study.**
(XLS)

## Abbreviations

CIS: Commonwealth of Independent States
DHS: Demographic and Health Surveys
UN IGME: United Nations Inter-agency Group for Child Mortality Estimation
